# Arsenic concentrations in local aromatic and high-yielding hybrid rice cultivars and the potential health risk: a study in an arsenic hotspot

**DOI:** 10.1007/s10661-017-5889-3

**Published:** 2017-03-24

**Authors:** Arifin Sandhi, Maria Greger, Tommy Landberg, Gunnar Jacks, Prosun Bhattacharya

**Affiliations:** 1 Department of Ecology, Environment and Plant Sciences, Stockholm University, Svante Arrhenius väg 20 A, SE-114 18 Stockholm, Sweden; 2 Division of Land and Water Resources Engineering, Department of Sustainable Development, Environmental Science and Engineering, KTH Royal Institute of Technology, Teknikringen76, SE-100 44 Stockholm, Sweden

**Keywords:** Aromatic rice, Arsenic, As uptake, Accumulation factor, Bangladesh, Food chain, Husk, Soil

## Abstract

The presence of high levels of arsenic (As) in rice fields has negative effects on the health of those consuming rice as their subsistence food. This study determined the variation in total As concentration in local aromatic rice (LAR) (*kalijira*) and two high-yielding varieties (HYVs) (BRRI dhan 32 and BRRI dhan 28) grown in paddy fields in Matlab, Bangladesh, an As hotspot with elevated As levels in groundwater. Mature rice grain samples and soil samples were collected from different paddy fields, and the As concentrations in both the de-husked grains and the husks of the three rice cultivars were analysed to identify the safest of the three cultivars for human consumption. The results showed that the total As concentration was higher (0.09–0.21 mg As kg^−1^) in the de-husked grains of LAR than in the husks, while the opposite was found for the HYV rice. Moreover, the As concentration in soil samples was 2 to 5-fold higher for the LAR than for the HYVs, but the As accumulation factor (AF) was lower in the LAR (0.2–0.4%) than in the HYVs (0.9–1%). Thus, LAR can be considered the safest of the three cultivars for human consumption owing to its low AF value. Furthermore, due to the low AF, growing LAR instead of HYVs in soils with slightly elevated As levels could help improve the food safety level in the food chain.

## Introduction

The presence of high levels of arsenic (As) in groundwater in Bangladesh and its negative impact on human health is one of the major environmental problems in the region (Smith et al. 2000). Due to the lack of a well-planned drinking water supply chain and safe surface water in rural areas, local inhabitants have no choice but to use As-contaminated groundwater for drinking and food preparation. The use of contaminated groundwater for drinking purposes is widely recognised as the major source of human exposure to arsenic. A number of recent studies also report that another major source of As exposure is through the regular consumption of rice cultivated and irrigated with As-contaminated groundwater (Williams et al. 2006; Sun et al. 2012). This is the prime cause of increasing concerns regarding the As content in food, as it is associated with carcinogenicity and a number of multiple adverse health consequences (e.g. skin lesions, genotoxic effects, cardiovascular diseases, etc.) (Ljung et al. 2011; Gilbert-Diamond et al. 2012).

Arsenic in the environment exists in both organic and inorganic forms and is influenced by pH and redox conditions (Zhao et al. 2010). The inorganic forms are more toxic than the organic forms regarding their impact on organisms (Greger et al. 2015). Crop plants contain mainly inorganic As forms (arsenate [As^V^] and arsenite [As^III^]). Arsenate is found in aerobic conditions, while arsenite is more prominent in paddy soil or submerged soil (Zhao et al. 2010). Rice also contains less toxic organic As species such as dimethylarsinic acid (DMA) and monomethylarsonic acid (MMA) (Meharg et al. 2009).

Rice has a higher tendency for As uptake than other cereals as it is grown in submerged soil conditions, where arsenite is more available than arsenate (Zhao et al. 2010; Begum et al. 2016). In Bangladesh, rice is one of the major cereal crops, taking up 75% of the total cultivated area and using 83% of the irrigation water (BBS 2010). It has been reported that rice grains from Bangladesh contain higher levels of inorganic As than rice grains from the USA and Europe and that approximately 80% of the As is in inorganic form (Williams et al. 2005; Ma et al. 2016). The As content in the marketed rice differs from the As content in rice from the field, as the marketed rice is a collection of grain from large parts of a country, while rice collected from a specific site mirrors the local As contamination (land and soil) (Meharg and Zhao 2012). The regulation for a maximum level of inorganic As in rice and certain foods was recently established in the European Union (EU), whereby the maximum value for inorganic As content in rice and rice-based products was decided at 0.20–0.30 mg kg^−1^ and forrice-based baby food at 0.10 mg kg^−1^ (Ankarberg et al. 2015). Conversely, no maximum As content in food has been established in Bangladesh to date. Besides local rice cultivars, other newly developed high-yielding varieties (HYVs) are also cultivated in Bangladesh, with as many as 57 HYVs having been launched so far by the Bangladesh Rice Research Institute (BRRI) (Hossain et al. 2013). A few of the local rice varieties (e.g. *kalijira*, *chinigura*) have a distinct aroma and attract a price premium for this aromatic feature. Compared to local aromatic rice (LAR), most of the HYVs require extensive irrigation for maturity and production. However, groundwater-based irrigation is considered one of the major pathways of As loading in agricultural soil types and crop plants, including rice (Meharg and Zhao 2012; Sultana et al. 2015).

To determine the magnitude of the As problem in rice, a number of investigations have been carried out in Bangladesh, most of which are either market-based surveys or pot-based experiments (Abedin et al. 2002; Meharg and Rahman 2003; Meharg et al. 2009). The As concentration in the de-husked grains and husks of HYV rice has been found to range between 0.59–0.81 and 1.7–6.2 mg kg^−1^, respectively (Rahman et al. 2007; Khan et al. 2010). As the husk tissue (the external part of the grain) comprises 21–30% of the whole weight of the rice grain, this implies that most of the As ends up in the husk. After rice has been de-husked, the husk fraction is then used as an animal feed ingredient. The As concentration in LAR cultivars has been found to range between 0.01 and 0.18 mg kg^−1^ and is thus lower than that in HYVs frequently grown in Bangladesh (Williams et al. 2006; Al-Rmalli et al. 2012). Considering this great variation in the total As content in rice grains between different cultivars, acquiring information about the total As content in rice cultivars (both HYV and local varieties) could be an important factor for the reduction of the As health risk from rice grown in As hotspots. A recent study has also found that the total As content in rice grains could be a satisfactory surrogate for the inorganic As species (arsenite) presence in the grains, since 60–90% of the total As content found in grains is in the form of arsenite (Ma et al. 2016).

In Matlab area in Bangladesh, there are approximately 13,000 drinking water tubewells. Approximately 80% of these have an As concentration in water exceeding 50 μg L^−1^, which is the maximum As limit in the drinking water standard for Bangladesh (von Brömssen et al. 2007). However, there is limited information available about the As concentration in rice cultivars grown in Matlab. To minimise the knowledge gap about As contamination and determine the potential health threat for local residents, it is important to investigate the level of As not only in drinking water but also in different varieties of rice grown using groundwater-based irrigation.

The aim of this study was to investigate the variation in As concentration within and between LAR and HYV rice collected from rice fields in Matlab. Three hypotheses were tested: (i) HYV rice accumulates more As than LAR (based on the fact that HYVs require more irrigation water than local rice varieties), (ii) both HYVs and LAR accumulate more As in the husk than in the de-husked grains and thus (iii) LAR is safer for humans to consume than HYVs in terms of As content.

## Materials and methods

### Location and rice cultivars

The study area, Matlab (23.3500° N 90.7083° E), is a subdistrict located approximately 60 km south-east of Dhaka, the capital city of Bangladesh. Matlab is one of several well-known As hotspots in south-eastern Bangladesh (von Brömssen et al. 2007). Rice cultivation is one of the major agricultural activities practised in all the villages of Matlab, due to suitable soil and climate conditions. A previous irrigation water survey by our research group found that the As concentration in irrigation water (203–349 μg As L^−1^) in Matlab was higher than that found in irrigation water available in non-contaminated areas of Bangladesh (Saha and Ali 2007). The study area was approximately 409 km^2^ and has a tendency to flood during the monsoon season every year. For the present study, samples of rice spikes with mature grains were collected from the underlying paddy soil at different locations within the study region.

### Rice and soil sampling

Nine rice fields with mature rice plants were selected for sampling. LAR (*Oryza sativa* var. kalijira), which is a slender-shaped, white-grain rice variety, was present in three of these fields. Another three fields contained the HYV *O. sativa* var. BRRI dhan 32, a medium-bold, white-grain rice, while the last three fields contained the HYV *O. sativa* var. BRRI dhan 28, a medium-slender, white-grain rice. In each field, three random surface paddy soil samples from 0 to 15-cm depth were collected using a hand spade. Mature rice panicles were randomly collected from three locations in each field, close to the area where the soil samples were collected.

### Sample preparation for analysis

#### Soil

The soil samples were cleared of plant debris and kept in a cold chamber until further analysis in the laboratory. Before analysis, the soil samples were dried at 60 °C for 72 h. The reason for drying at 60 °C was due to the fact that the As content in the samples could potentially evaporate at temperatures above 80 °C. The dry weight of the samples was then measured, and 0.20–0.42 g of soil samples were placed in a wet digestion block and digested in 7 M HNO_3_ to analyse the total As concentration in the soil samples (Bergqvist and Greger 2012; Stoltz and Greger 2006).

#### Rice

The collected mature rice grains were separated from the panicle, and external debris was removed. The grains were then de-husked by hand using tweezers, separated into a de-husked grain and a husk fraction. Both the de-husked grain and husk fraction were dried at 60 °C for 72 h. After drying, both fractions were wet digested in HNO_3_/HClO_4_ (7:3, *v*/*v*) for 19 h in a heating programme with the temperature increasing up to 225 °C (Bergqvist and Greger 2012; Frank 1976). Subsamples of the dried soil, de-husked grains and husks were dried at 105 °C for 24 h to recalculate the real dry weight for use in the calculation of the total As concentration.

### Analysis

The total As concentration in wet-digested soil samples was analysed using inductively coupled plasma optical emission spectrometry (ICP-OES; Thermo iCAP 7600). The total As concentration in the de-husked grains and husks was measured with atomic absorption spectrometry (AAS; Varian Spectr AA 55B vapour generation technique, VGA-77). For hydride generation, laboratory standard sodium borohydride (3%; Merck), sodium hydroxide (2.5%; EKA Chemicals) and hydrochloric acid (6 M; VWR International) were used.

### Calculations and statistical analysis

The arsenic concentration in whole grains was calculated from the As concentration in the de-husked grains and husks using the equation:

1 $$ {\left[\mathrm{As}\right]}_{wg}=\frac{\left({\mathrm{Weight}}_h\times {\left[\mathrm{As}\right]}_h\right)+\left({\mathrm{Weight}}_{dg}\times {\left[\mathrm{As}\right]}_{dg}\right)}{{\mathrm{Weight}}_h+{\mathrm{Weight}}_{dg}} $$

where *wg* denotes whole grains, *dg* de-husked grains and *h* husks.

The As accumulation factor (AF) was calculated to compare the As accumulation properties of the different cultivars at various soil As concentrations using the equation:

2 $$ \mathrm{AF}={\left[\mathrm{As}\right]}_{\mathrm{whole}\ \mathrm{grain}}/{\left[\mathrm{As}\right]}_{\mathrm{soil}} $$

The potential daily As intake of a 70-kg individual was calculated in accordance with Sun et al. (2012) using the equation:

3 $$ {\mathrm{As}}_{\mathrm{individual}}\ \left(\upmu \mathrm{g}\ \mathrm{As}\ {\mathrm{day}}^{-1}\right)={\left[\mathrm{As}\right]}_{\mathrm{de}-\mathrm{husked}\ \mathrm{grain}}\times \mathrm{consumption}\ \mathrm{rate}\ \left(\mathrm{g}\ {\mathrm{day}}^{-1}\right) $$

All calculations were performed with Microsoft Office Excel (1997–2003). Before the statistical analysis, the data were checked for normality where necessary. All values presented are the mean of three replicates, and the statistical significance of the differences was calculated with Tukey’s *t* test.

### Quality control

A standard reference material GBW07604 (GSV-3, poplar leaves, Institute of Geophysical and Geochemical Exploration, Langfang, China) was analysed following the same analytical procedure as was used to analyse the As content in de-husked rice grains and husks. The certified value for total As concentration in GBW07604 (0.37 ± 0.06 μg As g^−1^) gave an As recovery of 92.3%. The standard addition method was applied to the AAS measurements for compensating the matrix effect during analysis of the total As concentration. The standard addition method was also applied to measure the total As concentration in soil samples using ICP-OES.

## Results and discussion

**Fig. 1:**
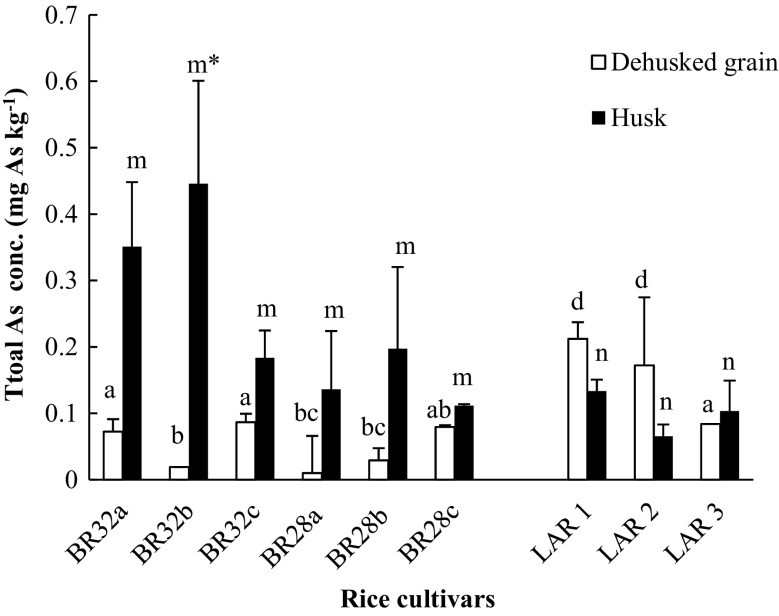
Concentration (mean ± SE, mg As kg^−1^) of arsenic (As) in de-husked grains (*white bars*) and husks (*black bars*) of high-yielding rice varieties (HYVs) (BRRI dhan 32 and BRRI dhan 28) and in local aromatic rice (LAR) (*kalijira*). *n* = 3. Different letters (de-husked grain (*a–d*), husk (*m–n*)) indicate significant differences between HYV and LAR samples (*p* < 0.05). Significant differences in As concentration among de-husked grain and husk for individual samples are also marked (*asterisk*)

**Fig. 2:**
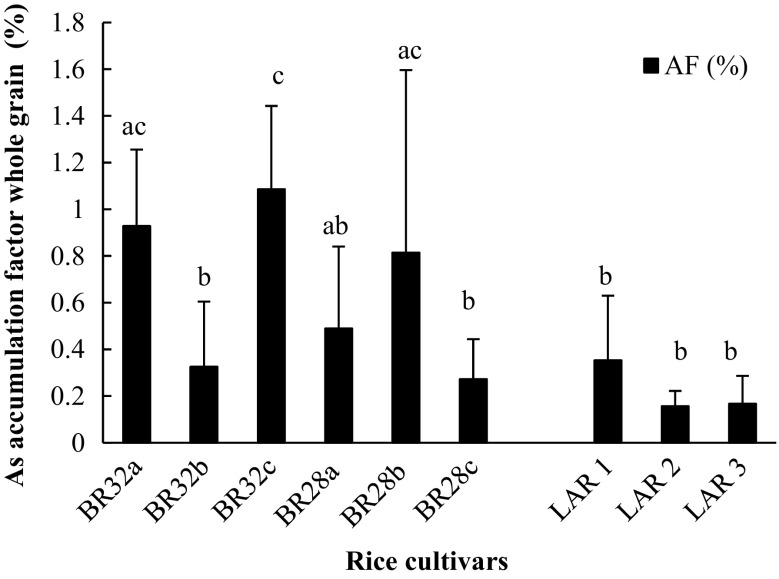
Accumulation factor (AF) (mean ± SE, %) of arsenic (As) in whole rice grains of high-yielding varieties (HYVs) and local aromatic rice (LAR). *n* = 3. Different letters (*a–c*) show significant differences between HYV and LAR (*p* < 0.05)

**Fig. 3:**
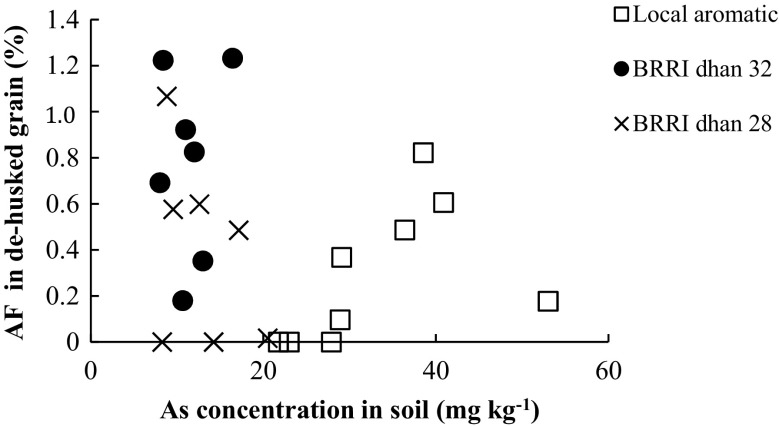
Correlation between arsenic (As) accumulation factor (AF) (%) of de-husked rice grain (BRRI dhan 32, BRRI dhan 28 and *kalijira*) and total As concentration (mg kg^−1^) in the soil

The data showed that the total As concentration in whole grain (de-husked grain + husk) did not differ significantly between the HYVs and LAR, even though the soil As concentration in LAR fields was 2 to 5-fold higher than in fields with the HYV cultivars (Table 1). All soil samples collected contained some As, with the total concentration ranging between 5.7 and 33 mg As kg^−1^ (Table 1). The total As concentration in soil of both LAR and HYV rice types was in a similar range to that reported in soil in previous studies (Meharg and Rahman 2003; Islam et al. 2004). A recent study has shown that the As content of soil has a significant effect on As uptake in rice roots and transfer to grains (Lin et al. 2015). Similarly, our data showed that the As concentration in soil may have influenced the As concentration in the rice grain, especially in the husk part (Table 1, Fig. 1). Therefore, to compare the ability of HYVs and LAR to accumulate As, we calculated the AF of all the rice varieties (Fig. 2). Although the As AF varied greatly between samples within each rice variety, there was still a tendency for the HYV rice to have a higher AF than the LAR variety. This difference was especially noticeable at low As concentrations in the soil (Fig. 3). In this study, the As concentration of the soil was 2 to 5-fold lower for the HYVs than for LAR-type paddy fields. However, based on the AF value of rice varieties, it was predicted that the As uptake by HYV rice cultivars would be higher than in the LAR type in highly As-contaminated fields. When grown in similar levels of As-contaminated fields, it can also be anticipated that the accumulation of As in HYV grains would be higher than in LAR varieties, due to their higher AF values (Fig. 2). Therefore, our first hypothesis, that HYV rice accumulates more As than LAR, was partly confirmed, based on their AF values.

**Table 1:** Arsenic (As) concentration (mean ± SE, mg kg^−1^) in soil and in whole grains (husk + grain) of high-yielding varieties (HYVs) and local aromatic rice (LAR) collected in nine paddy fields in Matlab, Bangladesh (*n* = 3)

Type	Rice cultivar	Rice sample code	As concentration (mg kg^−1^)	
			Soil	Whole grains
HYV				
	BRRI dhan 32	BRRI 32 a	13.7 ± 1.0 a	0.11 ± 0.02 s
	BRRI dhan 32	BRRI 32 b	16.8 ± 2.9 b	0.04 ± 0.02 t
	BRRI dhan 32	BRRI 32 c	9.1 ± 0.7 c	0.10 ± 0.02 s
	BRRI dhan 28	BRRI 28 a	5.7 ± 2.3 c	0.05 ± 0.01 t
	BRRI dhan 28	BRRI 28 b	9.4 ± 0.1 c	0.08 ± 0.05 st
	BRRI dhan 28	BRRI 28 c	14.7 ± 1.1 ab	0.02 ± 0.01 t
LAR				
	Kalijira	LAR 1	33.0 ± 4.7 d	0.10 ± 0.06 s
	Kalijira	LAR 2	30.2 ± 3.7 d	0.13 ± 0.07 s
	Kalijira	LAR 3	28.4 ± 6.7 d	0.08 ± 0.07 st

Different letters (a–d, soil; s–t, whole grain) within columns indicate significant differences (*p* < 0.05)

Our second hypothesis stated that both HYVs and LAR accumulate more As in the husks than in the de-husked grains. Analysis of As concentration in samples from all three rice varieties (HYVs and LAR types) partly confirmed this hypothesis, showing 1.3 to 15-fold higher As concentrations in the husk than in the de-husked grain of the HYV rice types (Fig. 1). Similar results have been presented by Rahman et al. (2007) and Khan et al. (2010), who found that the total As concentration differed by a factor of 3–9 between husks and de-husked grains. However, our investigation found the opposite of that trend in two out of three LAR varieties sampled, where the total As concentration in the de-husked grains was up to 2-fold higher than in the husks (Fig. 1). This difference could be explained by the fact the LAR rice husks are relatively strongly attached to the grain compared with HYV types and that the whole de-husking process was performed manually (using hands and tweezers) in the laboratory. Consequently, since the grains were not polished as the market rice, the As content in LAR variety rice grain was increased. Thus, the assumption regarding As distribution in different rice varieties as husk > bran-polished rice > brown rice > polished rice (Rahman et al. 2007) was not validated by the As concentrations in the LAR samples in our study. Two previous investigations regarding As content in rice also found that the As concentration in grain of LAR (kalijira, same variety we have investigated) was 0.013 and 0. 18 mg kg^−1^, respectively, and was different as they were collected from different regions (Williams et al. 2006; Al-Rmalli et al. 2012). Compared with those studies, the As concentration was higher in the de-husked grain part of the LAR variety (kalijira) in our investigation. The main reason that could have played a significant role is that our LAR samples were collected directly from an As hotspot area.

Our third hypothesis stated that the LAR rice variety is safer for human consumption than HYVs because of the lower As content. The results showed that the LAR specimens accumulated more total As in the de-husked grains than in either the husks or the de-husked grains of the HYV rice (Fig. 1). Calculations based on Sun et al. (2012) also showed that the LAR variety transferred 3 to 4-fold more total As to the human food chain than the HYV rice (Table 2), indicating that the HYVs were the safer rice compared to the LAR variety. However, the AF value was higher in HYVs (BRRI dhan 32 and BRRI dhan 28) grown in soils with lower As concentrations than in LAR paddy fields, suggesting that LAR is safer for human consumption than HYVs due to its low AF value. The soil texture and the irrigation pattern were similar for these rice fields. This difference between AF values of rice grain could be the outcome of different redox conditions of the soil. A recent study found that oxic and anoxic conditions play an important role for As uptake in rice grains (Wu et al. 2017). It should be borne in mind, however, that our calculations were based on rice samples directly collected from the field and that the As concentration in the steamed or cooked grain needs to be further investigated for health risk assessment purposes; a recent study has found that the procedure of rice cooking could also influence the As content in rice (Basu et al. 2015). Another study on Bangladeshi inhabitants showed a significant positive correlation between cooked rice intake and urinary total As content (Melkonian et al. 2013). Therefore, the As concentration in the cooked grain of LAR cultivars grown in an As hotspot is an important research question that needs to be investigated in the future.

**Table 2:** Distribution (mean ± SE, %) of total arsenic (As) between husks and de-husked grains of high-yielding varieties (HYVs) and local aromatic rice (LAR) and calculated total human intake of arsenic from the rice cultivars in a 70-kg individual (*n* = 3)

Rice type	Rice cultivar	As distribution (%)		Uptake in human body (μg As day^−1^)
		De-husked grain	Husk	
HYV	BRRI dhan 32	47.8 ± 7.32 abd	52.2 ± 6. 74 abcd	30
HYV	BRRI dhan 28	34.6 ± 20.7 ab	65.3 ± 19.9 cd	20
LAR	Kalijira	63.2 ± 17.1 acd	39.1 ± 17.9 abcd	80

Different letters show significant differences at *p* < 0.05. Average rice consumption rate for adults is 500 g day^−1^

## Conclusions

It has already been documented that the application of As-rich irrigation water on rice is one anthropogenic factor accounting for a high As content in paddy soil, thus leading to a high As accumulation in rice (Abedin et al. 2002). Generally, irrigation is mainly applied in earlier growth stages of rice cultivation (e.g. vegetative and reproductive stages). However, our investigation (rice sample collection) was conducted during the ripening stage of rice cultivation, and irrigation was not applied during that time. It can be concluded that to minimise the As-related health risk from rice consumption in an As hotspot, besides irrigation water management, both the As content in soil and the As AF value of different rice varieties need to be considered. Therefore, screening for rice varieties like LAR and other similar varieties with a low AF can be recommended. Knowledge of the As AF value of different hybrids and local rice cultivars could be considered as an effective and useful tool for identifying safe rice cultivars that could grow on soils with slightly elevated As concentrations. This would reduce the As concentration in the human food chain, thus improving food safety levels for humans.
